# Beetles in bamboo forests: community structure in a heterogeneous landscape of southwestern Amazonia

**DOI:** 10.7717/peerj.5153

**Published:** 2018-07-03

**Authors:** Jennifer M. Jacobs, Rudolf von May, David H. Kavanaugh, Edward F. Connor

**Affiliations:** 1 Department of Biology, San Francisco State University, San Francisco, CA, United States of America; 2 Museum of Zoology, Department of Ecology and Evolutionary Biology, University of Michigan—Ann Arbor, Ann Arbor, MI, United States of America; 3 Department of Entomology, California Academy of Sciences, San Francisco, CA, United States of America

**Keywords:** Coleoptera, *Guadua* bamboo, Terra firme, Pitfall traps, Biodiversity

## Abstract

Amazonian bamboo forests dominated by large woody bamboo plants in the genus *Guadua* cover approximately 180,000 km^2^ and represent a key resource for many organisms. In southwestern Amazonia, native bamboo forests differ in structure, biodiversity, and growth dynamics from other forest types in the region. However, with the exception of a few species in which habitat specialization or a strong habitat association has been demonstrated, little is known about how bamboo forests influence animal community structure. In an effort to understand more about the animal assemblages associated with Amazonian bamboo forests, we characterized the structure of ground-dwelling beetle assemblages living in bamboo forests and adjacent terra firme forests in a lowland rainforest site in Peru. We conducted intensive pitfall trap surveys in 13 bamboo habitat patches and 13 adjacent terra firme habitat patches to determine if there were differences in the abundance and richness of beetle species in these two habitat types. Additionally, given that southwestern Amazonia experiences distinct dry and wet seasons, we conducted our study during the dry and wet season of one year to account for differences in seasonality. We found a distinct beetle assemblage associated with each forest type, and identified a set of dominant species that significantly contributed to the distinctness in beetle community structure between bamboo and terra firme forest. The terra firme forest had a greater number of rare species than the bamboo forest. Several beetle species exhibited a strong association with the bamboo forest, including a large species of Scarabaeidae that appears to be specializing on bamboo. We also found marked differences in beetle assemblages between dry and wet seasons. Our results support the prediction that beetle community structure in bamboo forest differs from that of terra firme in terms of species richness, abundance, and composition. Bamboo-associated animal communities require more exploration and study, and must be included in regional conservation plans seeking to protect entire animal communities in southwestern Amazonia.

## Introduction

Bamboo forests in southwestern Amazonia cover approximately 180,000 km^2^ and represent the largest bamboo-dominated forest in the Neotropics (Nelson, 1994; Griscom & Ashton, 2003). These forests are primarily dominated by woody species in the genus *Guadua*, a native bamboo that has been present in the region since the pre-Holocene as suggested by fossil evidence (Olivier et al., 2008), or perhaps earlier periods as suggested by recent phylogenetic studies (Ruíz-Sánchez, 2011). Many animal species use bamboo habitat for shelter, foraging, reproduction, or a combination of purposes (Emmons & Feer, 1990; Louton, Gelhaus & Bouchard, 1996; Kratter, 1997; Dunnum & Salazar-Bravo, 2004; Lebbin, 2007; von May et al., 2008; von May et al., 2009a; von May et al., 2009b; von May et al., 2010; Jacobs & von May, 2012; Jacobs, von May & Ratcliffe, 2012), although most of these species also live in other forest types and only a few are considered to be bamboo specialists (Kratter, 1997; Davidson, Arias & Mann, 2006; Davidson et al., 2006; Lebbin et al., 2007).

The dynamics of bamboo forests is relatively well understood, but little is known about the structuring of animal communities associated with bamboo habitat. Most bamboo in southwestern Amazonia grows in patches ranging from less than a hectare up to tens or even thousands of hectares (Griscom & Ashton, 2006). Those patches are typically surrounded by upland terra firme or, less frequently, by floodplain forest. Bamboo patches might flower synchronously and then die over large areas, and bamboo plants may or may not recolonize the same areas (Nelson, 1994; Griscom & Ashton, 2003; Silman, Ancaya & Brinson, 2003; Nelson & Bianchini, 2005; Griscom & Ashton, 2006; Olivier, 2007; Olivier, 2008). Thus large patches of bamboo may blink in and out of existence over time and space, presenting a dynamic, relatively short-lived resource for other organisms. In this context, it is relevant to investigate how animal communities are structured in bamboo forest compared to neighboring forest types. This question can be empirically addressed by conducting one or more surveys of species richness, composition and abundance in bamboo forest and neighboring forest habitat.

In an effort to understand more about animal communities associated with bamboo forests in southwestern Amazonia, our primary goal in this study was to quantitatively characterize the structure of ground-dwelling beetle assemblages living in bamboo forest and compare them with those of the adjacent terra firme forest. We selected beetles as focal taxa because they are taxonomically and trophically diverse, play vital roles in ecosystem maintenance, serve as an important food source for many vertebrates, and are relatively easy to collect (Didham, 1997; Ward & Larivière, 2004). Previous inventories of particular beetle taxa in a variety of habitats in the southeastern portion of the Peruvian Amazon have included minimal sampling in bamboo forests (Pearson & Derr, 1986; Erwin, 1997; Larsen, Lopera & Forsyth, 2006).

Until recently, with the exception of a small number of herbivorous beetles that are known to specialize on the *Guadua* plants themselves (T Erwin, pers. comm., 2007; Anderson, 2008) and a large dynastine beetle strongly associated with bamboo (Jacobs, von May & Ratcliffe, 2012), it was not known if other beetle species are closely associated with bamboo forest as a primary habitat, and whether distinct assemblages of beetles are associated with bamboo forests compared to the adjacent terra firme. Because of the structural differences between these two habitats (Griscom & Ashton, 2003; Griscom & Ashton, 2006), and the evidence of bamboo specialists in various animal taxa (see summary in Jacobs & von May, 2012), we hypothesized that beetle assemblages in bamboo forest would differ from those of terra firme. We predicted that some beetle species would show preferences either for bamboo or terra firme. Additionally, because of seasonal rainfall variation in southeastern Peru, we expected to find differences in beetle community structure between dry and wet seasons. Based on previous findings regarding temporal patterns of insect communities in Neotropical rainforests (Janzen, 1973; Pearson & Derr, 1986; Richards & Windsor, 2007), we hypothesized that beetle species richness and abundance would be greater in the wet season compared to the dry season.

## Materials and Methods

### Study site

**Figure 1:**
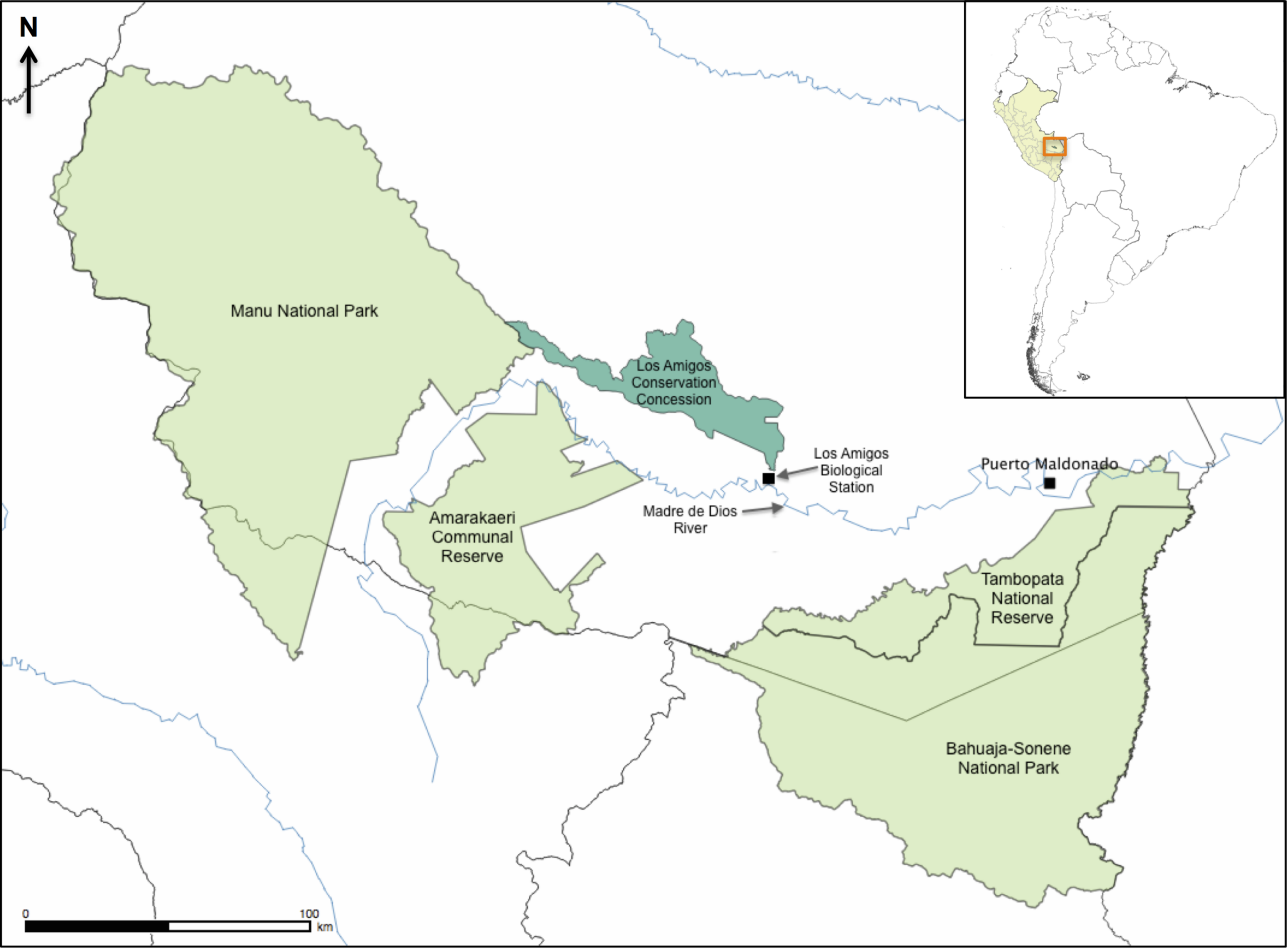
Map of southeastern Peru showing the location of the study area and other protected areas. All collecting took place near Los Amigos Biological Station, which is found on the southernmost border of the Los Amigos Conservation Concession. The other natural protected areas are Manu National Park, Amarakaeri Communal Reserve, Tambopata National Reserve, and Bahuaja-Sonene National Park. The inset shows the location of the study area in South America (Peru highlighted in yellow; area shown in detail in orange polygon). Map by Rudolf von May, made with QGIS (https://qgis.org/en/site/).

We conducted this study at the Los Amigos Biological Station (12°34′07″S, 70°05′57″W; 270 m elev.), in Manu province, Madre de Dios region, southeastern Peru (Fig. 1). The Los Amigos Biological Station (hereafter referred to as LA) covers approximately 1,000 ha and borders the Los Amigos Conservation Concession, which covers 145,918 ha of undisturbed lowland rainforest including several forest types (von May et al., 2010). Terra firme forest is the dominant habitat in the study area whereas the bamboo habitat occurs in patches of different sizes embedded in terra firme habitat. Annual rainfall is variable and ranges between 2,700 and 3,000 mm, and >80% of the rainfall in this region occurs during the October-May wet season. The dry season, which usually occurs between June and September and has precipitation below or around 100 mm/month, is relatively cooler than the wet season (http://www.atrium-biodiversity.org/, accessed on 10 April 2014). The mean annual temperature ranges between 21°C and 26°C (Pitman, 2008).

### Design of study

We selected four beetle families for analysis: Scarabaeidae, Carabidae, Histeridae, and Curculionidae. These families represent the majority of the individuals captured in our preliminary sampling. These families also span several functional groups, and include relatively large species, which simplifies curation and identification (Grimbacher & Stork, 2009). In addition, we targeted ground-dwelling and understory taxa from these families that would potentially be caught with pitfall traps. In focusing on these families, we were attempting to collect information on decomposers, fruit, seed, and humus eaters, generalist ground foragers, and generalist predators.

We used a paired sampling design with 13 sites, each consisting of one patch of bamboo and an equal sized adjacent area of terra firme. We followed a proportional sampling approach (Schoereder et al., 2004) in which sampling effort was standardized with respect to patch area for bamboo forest and an equal area for terra firme (one pitfall trap/hectare). Thus, depending on the area of the patch, bamboo patches had different numbers of traps. The terra firme forest received the same number of traps as their “paired” bamboo patches. We sampled each site during two periods in the dry season (July and August 2006) and two periods in the wet season (January and March 2007) to evaluate the effect of seasonality.

### Site selection

**Figure 2:**
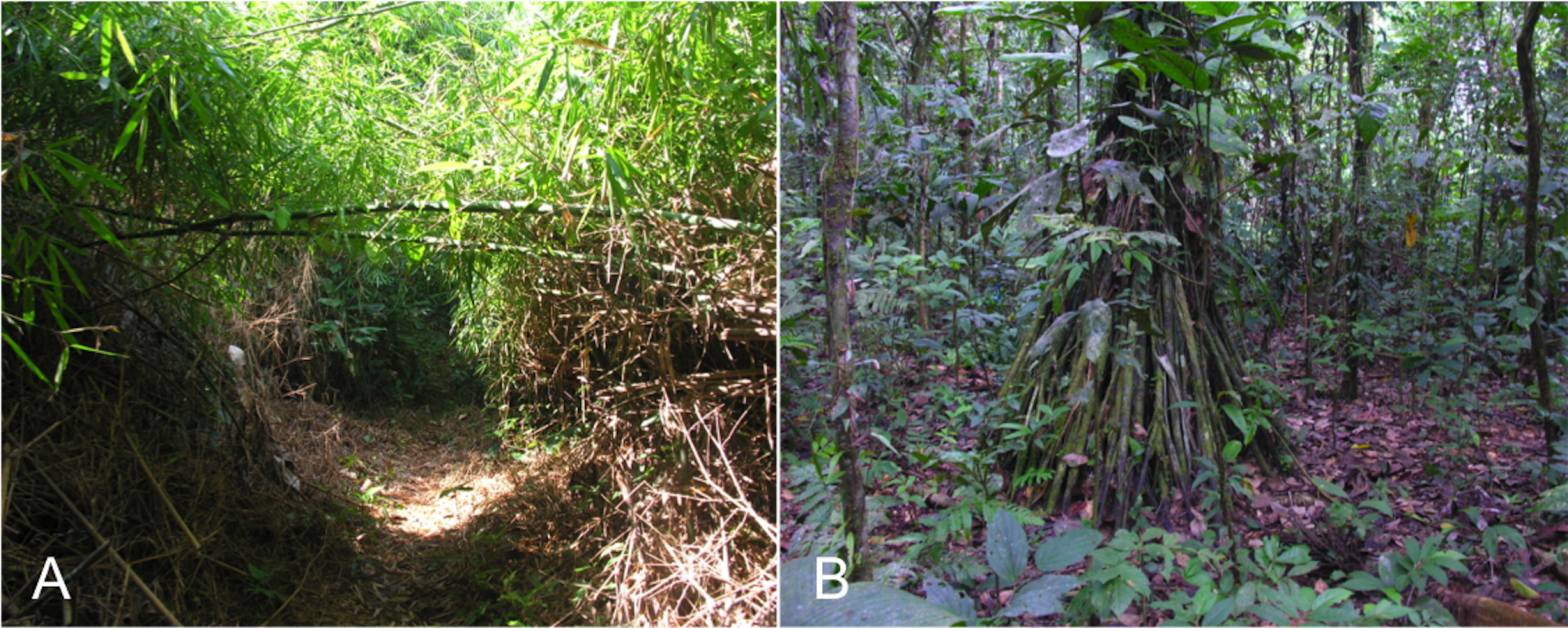
Views of a dense patch of *Guadua weberbauri* bamboo habitat with trail cut through patch (A) and of the terra firme habitat (B) studied at Los Amigos Biological Station, Peru. Photographs by Jennifer M. Jacobs.

**Figure 3:**
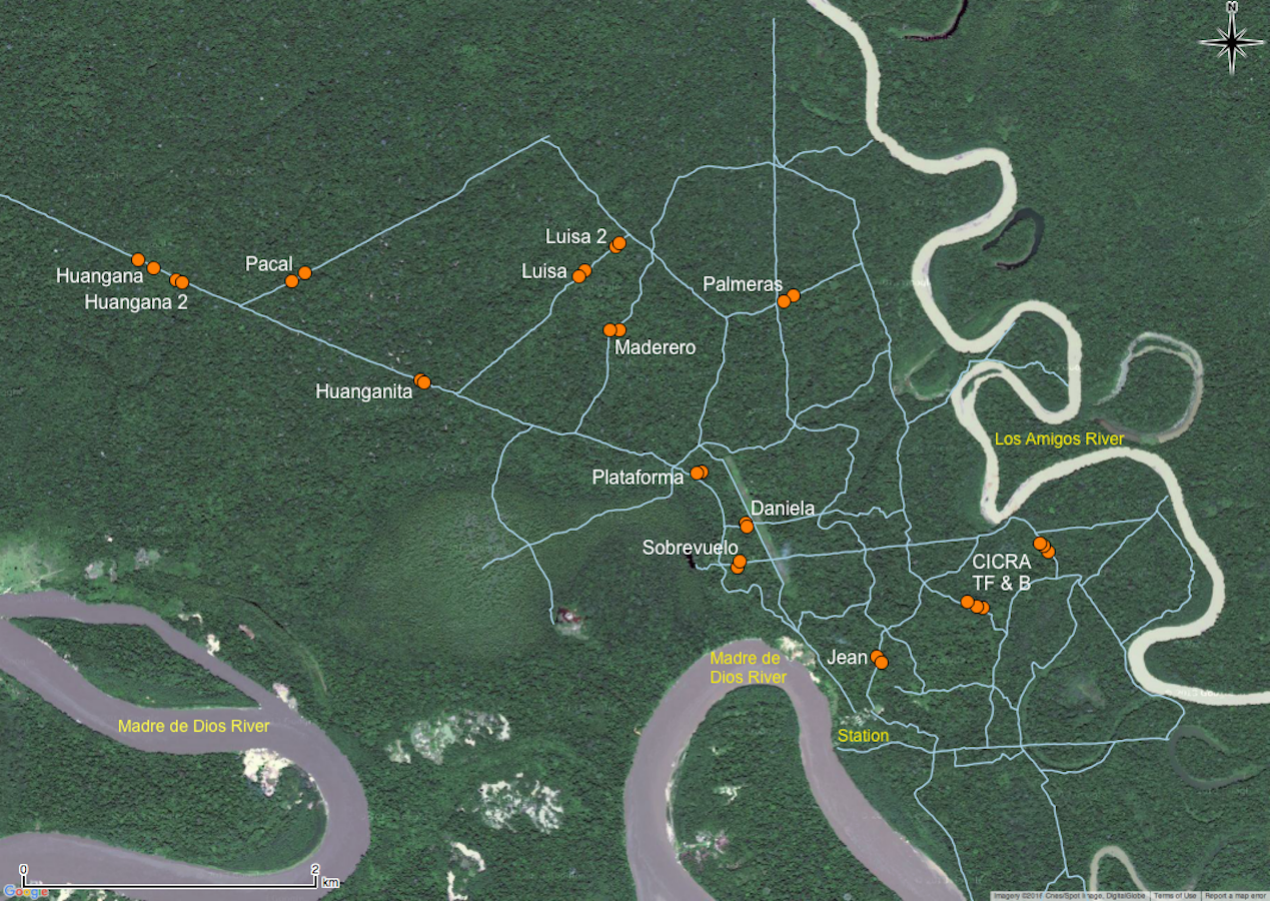
Map of the study area showing the general location of trap arrays (orange circles). The name of each trap array is noted in white and the trail system of Los Amigos Biological Station is shown in light blue. Scale bar = 2 km. Source of background and trail system layers: Google Satellite and Amazon Conservation Association.

We selected bamboo patches that contained dense stands of *Guadua weberbaueri*, few other plants, and leaf litter almost entirely composed of *G. weberbaueri* leaves to define a sample patch (Fig. 2). However, some patches also contained a second bamboo species, *Ichnanthus breviscrobs*. Though a few other intermittently distributed bamboo species occur at LA, the upland terraces are dominated by *G. weberbaueri* (Olivier, 2007). We selected sampling sites using Landsat images and vegetation maps used by previous researchers (Lebbin, 2007; M Tobler, pers. comm., 2006), and ground-based investigations along the entire trail system at LA (>100 km). We selected 13 dense patches of bamboo forest, which based on an initial power calculation, was sufficient for statistical analyses. Each bamboo patch and adjacent terra firme habitat (Fig. 2) was considered a site. We estimated the size of the bamboo patch using Arc GIS 9.1 and a pre-existing vegetation map of LA (D Lebbin, pers. comm., 2006). We also verified the GIS-derived measurements of bamboo patch size with ground observations. Every hectare within a selected bamboo patch, and adjacent terra firme, was allocated one pitfall trap, with patches ranging in size between approximately 1 and 25 ha. The general location of the 13 trap arrays is shown in Fig. 3, and the estimated patch area (ha), number of traps, and GPS coordinates are included in Table S1.

**Figure 4:**
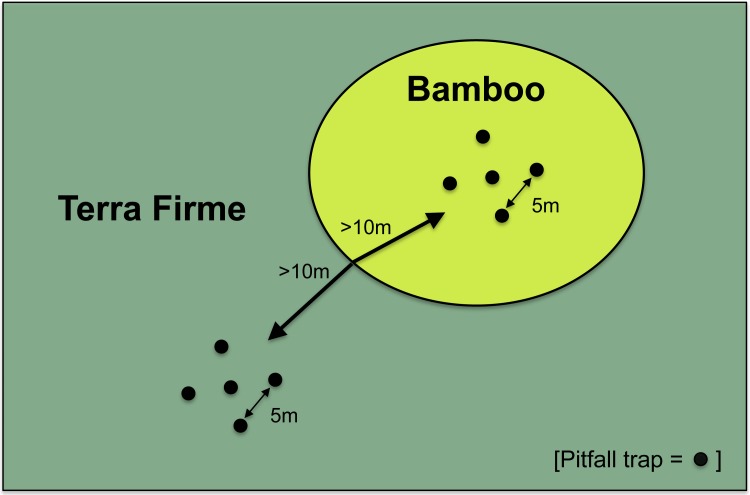
Sampling scheme of paired sites in bamboo and terra firme forest. Each pair of forest types received the same number of pitfall traps in the same array. Pitfall trap arrays were always greater than 10 m from the edge of a forest type and individual traps were always five meters apart.

### Sampling methods

We used un-baited pitfall traps to sample beetle communities. Pitfall traps are commonly used to catch surface-active invertebrates and are simple, inexpensive, and yield high numbers of specimens (Ward, New & Yen, 2001; Work et al., 2002). The advantage of using un-baited pitfall traps is that they represent a passive capture method to evaluate habitat preference as opposed to trapping with baits which may draw animals from greater distances, potentially outside the bamboo patch. Each pitfall trap consisted of two plastic 16-ounce containers (12 cm diameter) stacked together and inserted into the soil so that the top of the containers was flush with the ground. Traps were filled with approximately 0.125 L of a mixture comprised of 50% water and 50% ethanol (95% solution). A roof composed of palm leaves propped on a stick platform was placed 30 cm above each trap in order to prevent the traps from being flooded by rainwater or filled by falling leaves and other objects. The leaves covered the trap from above but allowed plenty of room for insects to enter laterally.

At each site, traps were placed 10–30 m into the bamboo patch, depending on the patch size. We then placed pitfall traps 5 m apart in either a linear or semi-circular array, depending on the shape, size and accessibility of the bamboo patch. Because *Guadua* forms extremely dense thickets and each plant is covered with large spines, it was impossible to access all locations within each patch. We always maintained a 5 m distance between traps regardless of the type of trap array. Our spatial array of traps was relatively similar to trap arrays used in studies by Barbosa & Marquet (2002), Driscoll (2005), and Baker & Barmuta (2006) (Fig. 4). We followed exactly the same procedure for placing traps in the adjacent terra firme. We recorded the location of trap arrays with a Garmin 76 Map GPS in the center portion of the trap array in each habitat at every site. In the largest patch of bamboo (∼25 ha), three separate transects of pitfall traps were established because the high density of bamboo culms made it impossible to place all 25 pitfall traps in one transect in the same location. Three separate transects were also established in the terra firme for that particular sampling site.

Pitfall traps were open for seven continuous days in both July and August of 2006 during the dry season, and seven continuous days in both January and March of 2007 during the wet season. Each seven-day period was considered a “sampling period”. During the interval between trap openings and closings, traps were monitored by visual inspection in the field for disturbance and functionality. If traps appeared dry, a small amount of ethanol was added. Traps were always examined following a large storm in case of flooding. For each sampling period, we used a total of 150 traps (75 for bamboo and 75 for terra firme), and with four sampling periods we employed a total of 600 traps. For the purpose of analyses and because we were interested in seasonal patterns, data from July and August were pooled as “dry season” and data from January and March were pooled as “wet season”.

### Sample processing

Following trap collection, specimens were cleaned, sorted, organized and preserved in jars containing 95% ethanol. Specimens were pinned, labeled, sorted to morpho-species, and entered into a database. Families, genera, species, and morpho-species were identified with help from a variety of taxonomic resources, including specialists at the California Academy of Sciences, Santa Barbara Natural History Museum, the Smithsonian Institution, and the University of Nebraska (see Acknowledgments). All other beetle specimens and arachnid specimens were kept and set aside for other researchers at the California Academy of Sciences and the Museo de Historia Natural San Marcos in Lima, Peru. Permits to conduct this work and collect specimens were issued by the Instituto Nacional de Recursos Naturales (INRENA), Peru (Research authorizations 053-2005-INRENA-IFFS-DCB, 23-2006-INRENA-IFFS-DCB, 67-2007-INRENA-IFFS-DCB, and 11-2008-INRENA-IFFS).

### Analyses

To initially compare patterns of beetle species richness as a function of sampling effort between terra firme and bamboo forest, we generated species accumulation curves (EstimateS 7.5; Colwell, 2005). We observed whether the confidence intervals of the curves overlapped to help determine if the communities of beetles in both habitats were different in terms of species richness. Although species accumulation curves are often used to determine the completeness of sampling when conducting an inventory (Longino & Colwell, 1997; Longino, Coddington & Colwell, 2002), they are also helpful in gross comparisons of communities in terms of diversity. Because we maintained exactly the same sampling effort in both habitats, we compared the curves directly and did not need to use richness estimators (Magurran, 2004). If the curve of one beetle community in either forest type was lower in species richness than the other forest type, we interpreted that result as a community pattern, not an artifact of under-sampling.

### Comparison of beetle assemblages between forest types

To statistically test for overall differences in mean species richness and abundance between the two habitats, we used a paired *t*-test to compare the species richness and abundance of beetles from all families at each pair of sites, while pooling data from dry and wet seasons. We used log transformations to normalize the data for these analyses, and *t*-tests were conducted in SPSS v. 11.0 (Chicago, IL, USA). We also used rank/abundance plots, also known as Whittaker plots, to illustrate contrasts or similarities in patterns of species richness and qualitatively assess the evenness of assemblages (Magurran, 2004; Gardner et al., 2007), to compare species abundance distributions in the two habitats. Prior to creating the Whittaker plots, we normalized our data with log-transformations and pooled data from dry and wet seasons.

To characterize the structure of beetle assemblages found in the bamboo and terra firme, we used species composition and abundance data collected in each habitat. However, we first used a Mantel test to test for a correlation between species composition and geographic distance among sites. We used a Bray–Curtis similarity matrix and coupled this with a matrix of all pairwise distances (in meters) between sites. We used an Excel spreadsheet integrated with PopTools ((http://www.poptools.org/)) to perform Mantel tests using 1000 randomizations of the distance matrix.

To compare beetle community structure in bamboo versus terra firme, while pooling across seasons, we conducted a one-way analysis of similarity (ANOSIM; Clarke & Warwick, 1994) using a Bray–Curtis similarity matrix. We used non-metric multidimensional scaling (nMDS; 100 restarts) plots to create graphical representations of ANOSIM results. The distance between points in nMDS plots is proportional to the compositional similarity of those points. Stress values of nMDS plots indicate the “goodness of fit” or quality of the test. Values beginning with zero (perfect fit) through 0.2 are considered effective for interpreting community data. All ANOSIM and nMDS analyses were conducted using a Bray–Curtis similarity matrix based on log-transformed data, with Primer 6.0 (Clarke & Gorley, 2006). We also used the application SIMPER (in Primer 6.0), considering each collecting site as a sample and each forest type as a group, to determine which species best characterized each habitat. This procedure initially calculates the species that account for 90% of the abundance of all species analyzed, and from that list, determines the percentages that each species contributes to the dissimilarity between any two groups.

We conducted a second set of multivariate analyses on each beetle family individually to determine if community structure for every family differed depending on forest type. We compared beetle species composition and abundance in bamboo to that of terra firme for each family (ANOSIM). Data were pooled across seasons.

To compare patterns of abundance or presence/absence of individual species between forest types, we created matched rank-occurrence plots with all species that were represented by at least five individuals. We applied this criterion because it is common that samples of tropical forest invertebrates contain many uncommon species and a substantial percentage of singletons (Coddington et al., 2009). This graphical method allowed us to visualize the degree of overlap between forest types for every species, and illustrate which species were absent, rare, or common in our collection (Longino & Colwell, 1997; Delsinne et al., 2008); the exclusion of rare species allowed us to better address differences driven by the most abundant species in the community.

### Comparison between wet and dry season

We tested for differences in overall mean species richness and abundance between the dry and wet seasons, using paired *t*-tests. To compare species abundance distributions between dry and wet season, we created Whittaker plots. We identified and ordered the ten most abundant species in each season to compare the identity and rank of the most abundant species during the dry and wet seasons. For both analyses, we pooled data from bamboo and terra firme and used log-transformed data.

To understand how species composition and abundance differed between dry and wet seasons, each within bamboo and terra firme, we used a variety of analyses. To test for seasonal differences in abundance within each forest type, we calculated the difference in number of individuals collected between dry and wet season at all sites for bamboo forest and for terra firme. We then applied a paired *t*-test to determine which forest type exhibited a greater change in beetle abundance between dry and wet season (SPSS v. 11.0, Chicago, IL). To test for differences in species turnover between seasons in bamboo and terra firme, we performed a similar procedure. We calculated how many species were unique between the dry and wet seasons for each site in the bamboo forest and in terra firme. We then applied a paired *t*-test to the number of unique species in each forest type.

To further explore the effects of habitat and season on beetle community composition, we performed both one-way and two-way ANOSIM analyses. To test for differences in beetle community composition between forest types in the wet and dry seasons individually, we performed one-way ANOSIM tests and associated nMDS plots. To simultaneously compare the effects of forest type and season on beetle community structure, we ran a two-way ANOSIM test using forest type and season as factors (we did not create an nMDS plot for the two-way ANOSIM because it is difficult to interpret given the more complex structure of the two-factor similarity matrix on which the nMDS analysis is based). The patterns of community structure are best visualized by looking at the one-way nMDS plots for the dry and wet seasons individually. In addition, we created matched rank-occurrence plots to compare patterns of abundance and presence/absence for individual species in the dry and wet seasons. For ANOSIM tests, nMDS plots, and matched rank-occurrence plots, we used species represented by at least five individuals.

## Results

### Comparison of beetle assemblages between forest types

The beetle community in bamboo forest differed from that of terra firme in terms of species richness, abundance and composition. We collected a total of 190 species of beetles and 3,752 individuals in our target families. In bamboo forest we trapped 120 species of beetles and 1,539 individuals,whereas in the terra firme we trapped 141 species of beetles and 2213 individuals (Table S2). Forty-eight species were found only in bamboo forest, 69 species were found only in terra firme, and 72 species were found in both forest types. Overall, beetles from the family Scarabaeidae comprised the majority of all specimens captured, accounting for 83% of the total number of individuals and approximately 48% of all species captured. All families except Histeridae were more species rich in the terra firme by 38%, 33%, and 11%, respectively for Carabidae, Curculionidae, and Scarabaeidae. All families except Carabidae were more abundant in the terra firme by 50%, 49% and 45%, respectively for Curculionidae, Histeridae and Scarabaeidae (Tables 1 and 2).

**Table 1:** The number of species of beetle in each family, and in all families combined, collected in only bamboo forest, only terra firme forest, in both habitats, only in the dry season, only in the wet season, and both seasons. The last column lists the total number of species collected per family.

Richness	Bamboo only	Terra Firme only	Both habitats	Dry only	Wet only	Both seasons	Total no. species
Carabidae	7	12	6	7	15	4	25
Curculionidae	16	25	11	13	31	8	52
Histeridae	6	6	10	7	5	10	22
Scarabaeidae	19	26	45	14	29	47	91
All families	48	69	72	41	80	69	190

**Table 2:** The number of individuals from each beetle family, and all families combined, collected in bamboo forests, terra firme forests, dry season, wet season. The last column lists the total number of individuals collected within each family and all for all families combined.

Abundance	Bamboo	Terra Firme	Dry	Wet	Total no. individuals
Carabidae	51	52	16	87	103
Curculionidae	62	93	71	84	155
Histeridae	153	228	204	177	381
Scarabaeidae	1,273	1,840	1,677	1,436	3,113
All families	1,539	2,213	1,968	1,784	3,752

**Figure 5:**
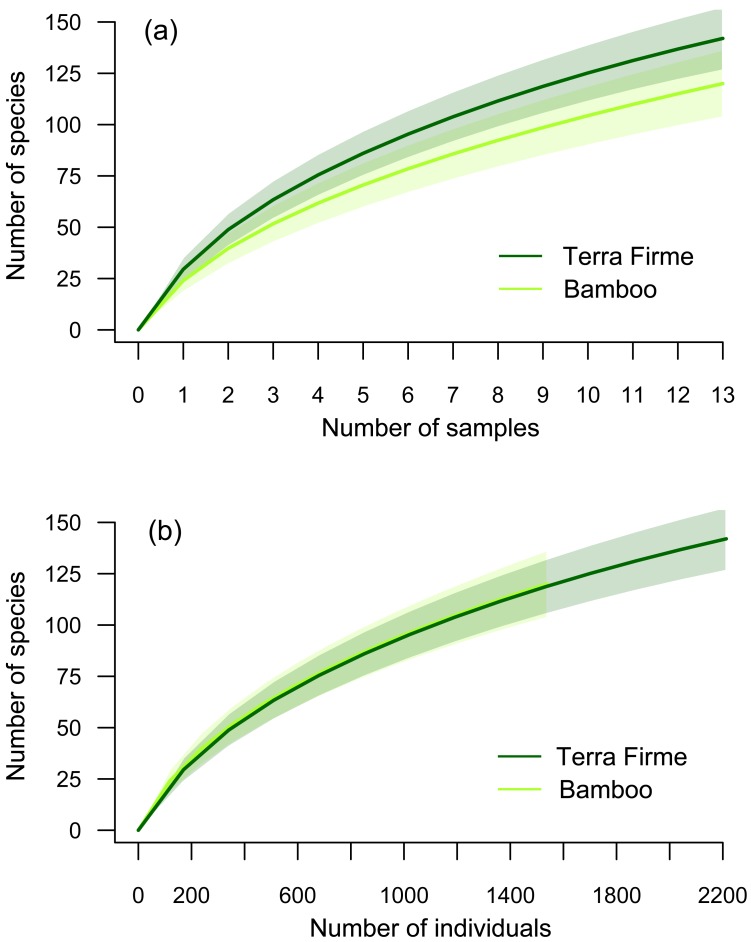
Species accumulation curves. (A) The number of species collected in bamboo and terra firme forests as a function of sampling effort and (B) the number of species collected in bamboo and terra firme forests as a function of number of individuals collected (shaded areas indicate 95% confidence intervals).

Overall, we found significant differences in the mean number of beetle species and individuals in bamboo versus terra firme using data pooled across seasons (*t*_12_ = 2.712, *p* = 0.019; *t*_12_ = 2.088, *p* = 0.059, respectively). The species accumulation curves illustrate that the number of species captured in terra firme was higher than that of bamboo forest, but overlapping confidence intervals indicate that the curves did not differ substantially (Fig. 5A). The results shown in Fig. 5B would support the idea that, for a given number of individuals sampled in each habitat, the expected number of species would be the same. So the slightly greater number of species of beetles observed in terra firme arises because terra firme forest supports a higher abundance of beetles in these families.

**Figure 6:**
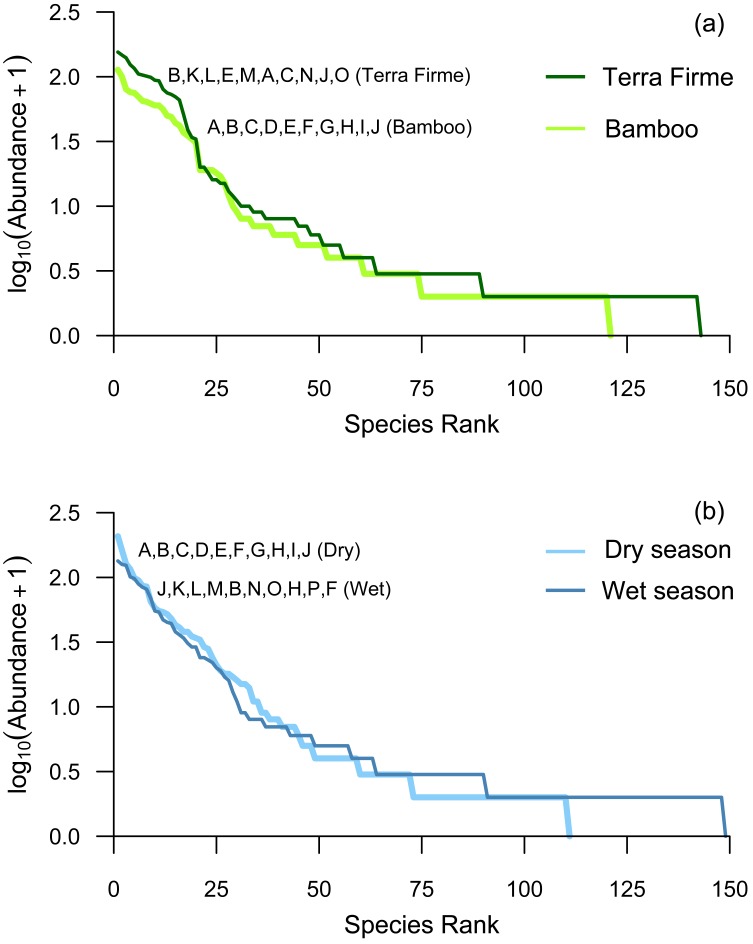
Whitaker plots comparing rank abundances between (A) forest types and (B) seasons. Species are ranked according to their abundances. Each letter represents one species, and the ten most abundant species are ordered. For each plot, one letter represents the same species, and a new letter is assigned for every new species. However, the letters are not constant for both plots in that species D in (A) may not be species D in (B). The lettered order illustrates that the ten most abundant species are not the same for bamboo and terra firme, and dry and wet seasons.

The species abundance distributions in the Whittaker plots for bamboo and terra firme (Fig. 6A) were similar, but terra firme exhibited higher abundances of the most abundant species and a surplus of rare species in comparison to bamboo forest. Though species abundance patterns were similar between bamboo and terra firme habitat, the order of the most dominant species differed between forest types. The ten most abundant species in order of rank abundance in bamboo forest (and displayed as letters on Fig. 6A) were not all the same species, nor were they in the same rank order as those in terra firme.

We did not detect any autocorrelation between differences in beetle assemblages and the distance between sites (Mantel test - Pearson *r* =  − 0.07888, *p* = 0.225). Therefore, we proceeded with multivariate analyses of beetle community structure.

**Figure 7:**
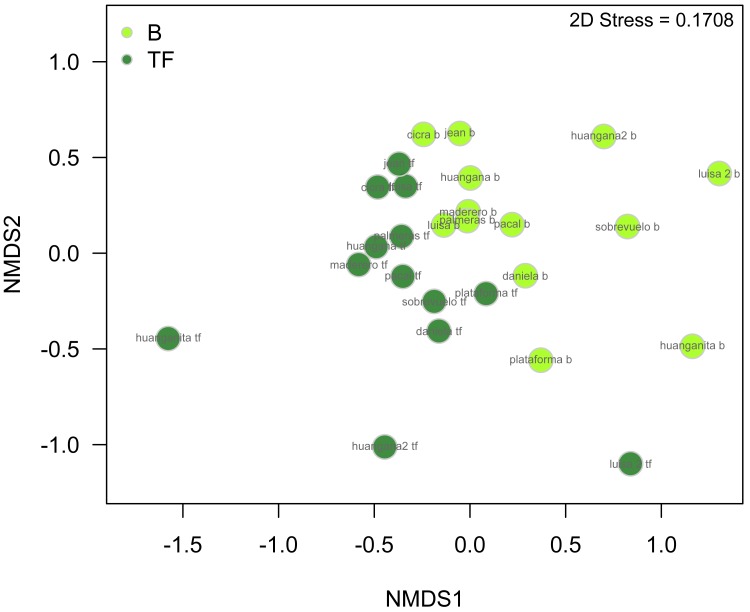
nMDS plot illustrating beetle community structure between bamboo and terra firme forest with data from all families and seasons pooled. The plot exhibits distinct communities of beetles in the two forest types with some degree of overlap between assemblages. Distance measure = Bray–Curtis. Legend: B, Bamboo forest; TF, Terra firme forest.

We found significant differences in beetle assemblages between bamboo and terra firme pooling across seasons (ANOSIM Global *R* = 0.160, *p* = 0.001). An nMDS plot (Fig. 7) also suggests the presence of two distinct beetle communities in the bamboo and terra firme, though one terra firme sample from one of the smallest sites (1 ha) is clearly an outlier. A set of 24 beetle species are largely responsible for the dissimilarity of beetle community composition between bamboo and terra firme (Table 3). Four of these species were very abundant only in bamboo forests, and eight other species were very abundant only in terra firme. The remaining 12 species were collected in both habitats but in varied abundances.

**Table 3:** Beetle species contributing 90% of the total number of individuals collected in each habitat (SIMPER –PRIMER 6.0). The values represent the percent contribution of each species to the distinctness in beetle community structure between bamboo and terra firme forest. The largest numbers are associated with species that most greatly distinguish assemblages in one forest type or another.

Species	Bamboo	Terra firme
**Carabidae**		
*Odontocheila cayennensis*	0	1.89
**Curculionidae**		
*Curculionidae 13*	0	0.96
**Histeridae**		
*Omolodes A*	6.71	8.35
*Phelister B*	2.1	5.62
*Operclipygus C*	1.66	0
**Scarabaeidae**		
*Enema pan*	14.8	0
*Scybalocanthon C*	12.63	8.51
*Canthidium A*	9.59	3.04
*Onthophagus xanthomerus*	9.4	10.96
*Canthidium F*	6.1	2.33
*Canthidium gerstaeckeri*	5.28	3.31
*Dichotomius ohausi*	5.03	6.51
*Dichotomius batesi*	4.87	0
*Dichotomius nr. lucasi*	3.57	3.34
*Canthonella D*	3.05	0
*Scybalocanthon D*	2.06	5.2
*Canthidium nr. deyrollei*	1.66	2.75
*Dichotomius lucasi*	1.58	3.09
*Ateuchus C*	0	7.21
*Ateuchus D*	0	3.11
*Canthidium batesi*	0	7.84
*Ceratocanthinae A*	0	1.4
*Deltochilum nr. komerecki*	0	1.19
*Eurysternus nov. stigilatus*	0	3.72

**Figure 8:**
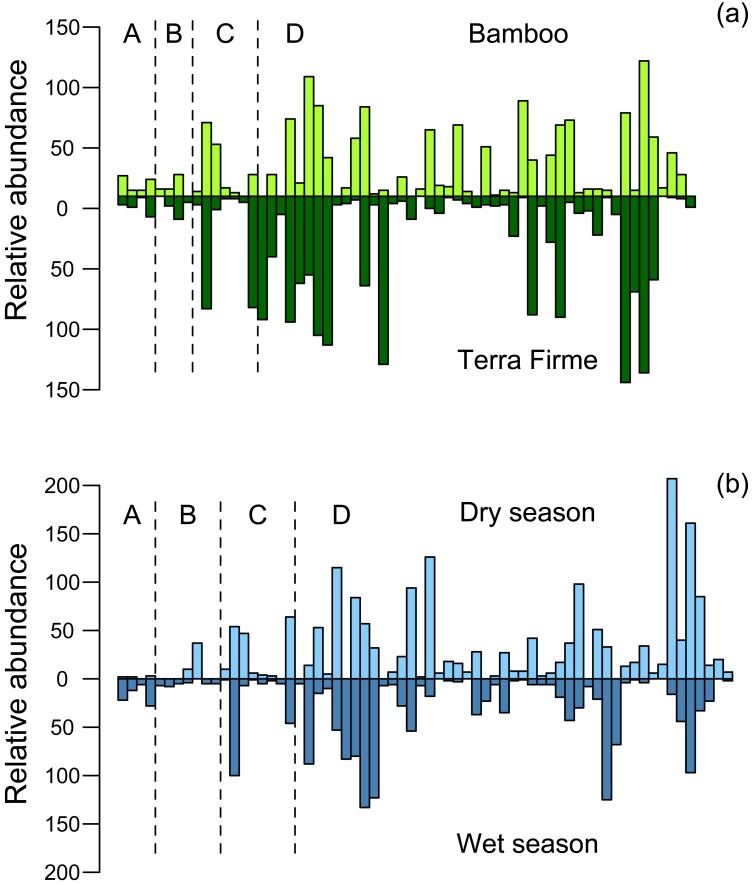
Matched rank/occurrence plots for (A) bamboo and terra firme forest and (B) dry and wet seasons. Each pair of positive and negative bars represents the relative abundance of one species in each forest type or each season. The letter “A” column represents beetles from the family Carabidae, “B” represents Curculionidae, “C” represents Histeridae and “D” represents Scarabaeidae.

For species represented by more than five individuals, matched rank-occurrence plots exhibit varying patterns of abundance and presence/absence in the two habitats (Fig. 8A). Many species exhibited greater abundances in only one forest type. Overall, we found a greater number of the most abundant species in the terra firme compared to the bamboo forest. Beetle species in the family Carabidae and Histeridae appear equally abundant in bamboo and terra firme whereas, Curculionidae and Scarabaeidae exhibited a greater number of species with higher abundances in the terra firme (Fig. 8A).

For two beetle families, community structure also differed between bamboo and terra firme. We detected significant differences in beetle assemblages between bamboo and terra firme for Scarabaeidae and Curculionidae (ANOSIM Global *R* = 0.190, *p* = 0.002; Global *R* = 0.154, *p* = 0.003, respectively), but not for Carabidae and Histeridae (Global *R* =  − 0.030, *p* = 0.785; Global *R* = 0.044, *p* = 0.187, respectively).

### Seasonality and forest type

With data from bamboo and terra firme pooled, overall mean species richness and number of individuals did not significantly differ between dry and wet seasons (paired—*t*_12_ = 0.907, *p* = 0.382; *t*_12_ = 1.619, *p* = 0.132, respectively). However, we collected 10% more individual beetles in the dry season and 35% more species in the wet season. Species abundance distributions were similar for the dry and wet season, but the rank-order of the most abundant species changed with the seasons and a greater number of rare species were detected in the wet season (Fig. 6B).

**Figure 9:**
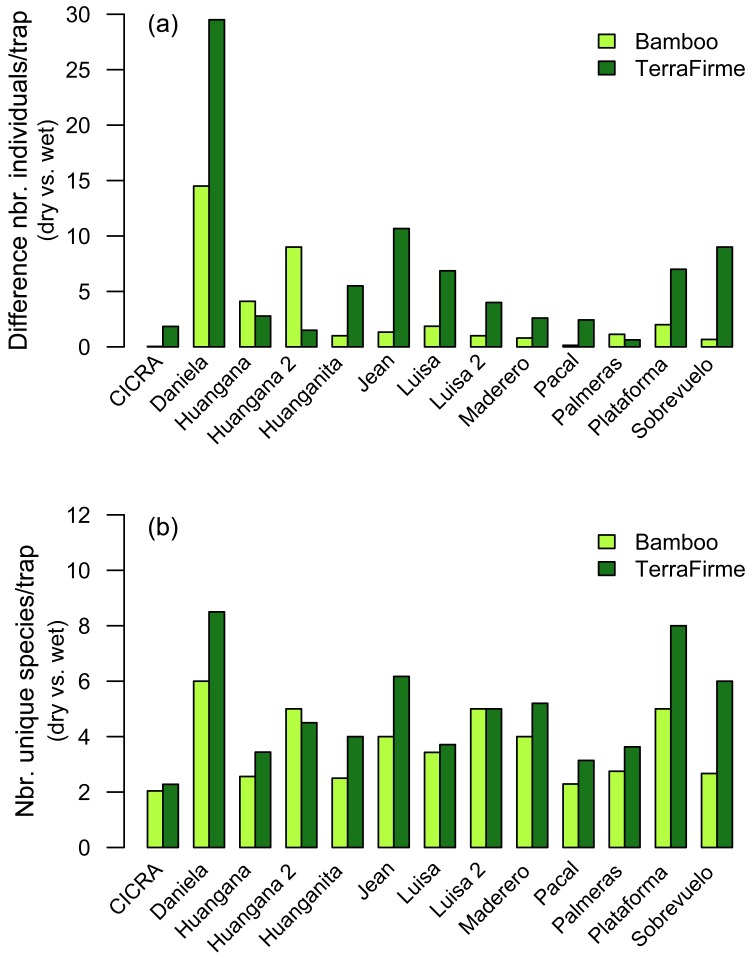
Plots expressing variation in (A) numbers of individuals/trap and (B) numbers of unique species/trap captured between dry and wet seasons, for bamboo forest compared to terra firme forest, at all sites. (A) Each bar represents the difference in number of individuals/trap captured between the dry and wet season in one forest type. (B) Each bar represents the number of unique (not shared) species captured/trap in the dry and wet season, in one forest type. In both (A) and (B), the pattern to observe is the difference between the paired bars, not that some bars are higher than others. Overall, there was more variation in the numbers of individuals captured and greater species turnover between dry and wet seasons in the terra firme forest compared to bamboo forest.

Seasonal differences in abundance and species turnover were greater in terra firme than in bamboo forest. We found a significant difference between the mean number of individuals collected in the dry versus wet seasons in terra firme, compared to that of bamboo forest, across all sites (paired—*t*_12_ = 2.951, *p* = 0.012). Similarly, we found a greater amount of species turnover between the dry versus wet season in terra firme compared to that of bamboo forest (*t*_12_ = 4.366, *p* = 0.001). A similar pattern was observed when expressing these results as mean difference in number of individuals/trap (*t*_12_ = 2.357, *p* = 0.036; Fig. 9A) and mean number of unique species/trap (*t*_12_ = 3.818, *p* = 0.002; Fig. 9B).

**Figure 10:**
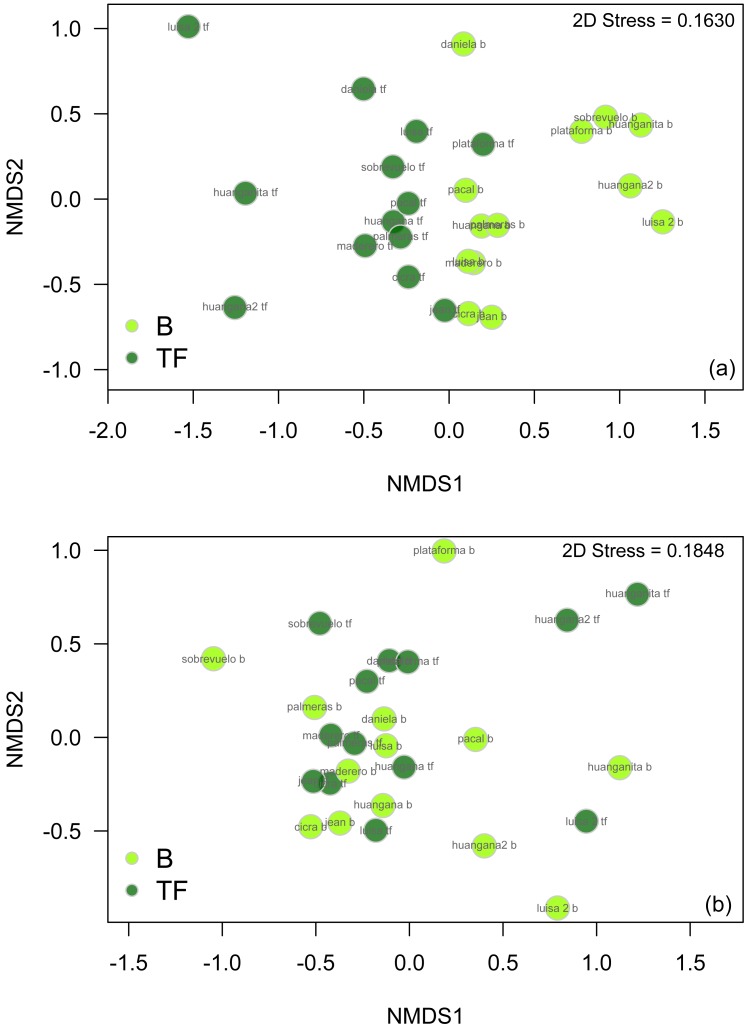
nMDS plots comparing beetle community structure between bamboo and terra firme forest in (A) the wet season only and (B) the dry season only. There is a greater distinction in beetle community structure between bamboo and terra firme forest in the wet season compared to the dry season.

Differences in beetle community structure between bamboo and terra firme was more pronounced in the wet season than in the dry season. We found significant differences in beetle community structure in the wet season (ANOSIM Global *R* = 0.259, *p* = 0.001, Fig. 10A), with less distinction between forest types in the dry season (ANOSIM Global *R* = 0.068, *p* = 0.070, Fig. 10B). However, the nMDS plot from the dry season illustrates some separation in beetle communities between bamboo and terra firme. One terra firme sampling point that appears to be a distinct outlier (the same site that is an outlier when examining data pooled among seasons, Fig. 7), may be influencing the results. The results of the two-way ANOSIM suggest that both forest type and season significantly affect beetle community structure, although the effect of seasonality was slightly stronger than that of forest type (season: Global *R* = 0.298, *p* = 0.001, forest type: Global *R* = 0.163, *p* = 0.001).

Matched rank-occurrence plots illustrate the variation in abundance for individual species, with more than five individuals captured, in the dry and wet seasons (Fig. 8B). Species in the family Carabidae were found primarily in the wet season, Curculionidae and Histeridae did not show strong seasonal variation, and we trapped more species of Scarabaeidae with higher abundances in the dry season. For a complete list of all species, where they were collected (forest type) and when they were collected (season) see Online Resource 1.

## Discussion

### Beetle assemblages in bamboo vs. terra firme

Our results support the prediction that beetle community structure in bamboo forest differs from that of terra firme in terms of species richness, abundance, and composition. We found a greater number of beetle species (17.5%) and individuals (43.8%) in terra firme compared to bamboo forest, but nonetheless found high beetle richness (120 species) and abundance in bamboo forest. Though relative abundance distributions were similar between bamboo and terra firme, each community was characterized by a distinct set of dominant species and terra firme had a greater number of rare species. In analogous study systems comprised of two naturally-occurring, contrasting, and adjacent habitat types, most often in the form of forest and savanna, researchers have reported distinct beetle communities for each habitat (Kotze & Samways, 2001; Spector & Aysama, 2003; Yu et al., 2007).

Our most striking results were the large number of beetle species found in bamboo, and that many species were only collected in bamboo forest and not in terra firme. Of the 120 beetle species we captured in the bamboo forest approximately 40% (48 species) were only collected in bamboo. Although further collecting might have yielded some of these species in terra firme, our results support the idea that bamboo is a rich habitat that contains a beetle community distinct from that of the adjacent terra firme. It is likely that some beetle species have evolved to specialize on bamboo forests as has been shown for other organisms (Kratter, 1997; Davidson, Arias & Mann, 2006; Kondo & Gullan, 2004; Lebbin et al., 2007).

Each beetle family exhibited different patterns of richness, abundance and composition within bamboo and terra firme. Because beetles from the Scarabaeidae constituted the majority of species in our collection (48%), and the majority of individuals (83%), diversity patterns of the scarabs were the most influential on the overall results. Out of the 24 most abundant species that best characterized either bamboo or terra firme, the greatest number of species (19) were scarab beetles (Table 3). All families except Histeridae were more species-rich in terra firme. All families except Carabidae (equally abundant in both forest types) were more abundant in terra firme.

We found significant differences in beetle community structure comparing bamboo and terra firme only for Scarabaeidae and Curculionidae. For Carabidae, it is possible that a low number of individuals (103) compared to the number of species (25), in addition to the high number of singleton and doubleton species in the carabid dataset (20), yielded nonsignificant results in terms of community structure between habitats. Data for the Carabidae family suggests that there are differences in beetle assemblages from bamboo and terra firme as there was a large turnover in species between bamboo and terra firme.

We collected many beetle species from every family that were found only in bamboo forest. Fifty-four percent of the carabid beetle species that we collected in bamboo were only from bamboo, followed by 59% for the Curculionidae, 38% for the Histeridae, and 30% for the Scarabaeidae. In addition, we found the greatest number of singletons and doubletons in the Carabidae and Curculionidae which may be influencing the high degree of observed habitat affinity compared to the more abundant families of Histeridae and Scarabaeidae. Carabid and curculionid beetles may also be more sensitive to forest type. Additional, and more targeted sampling is necessary to elucidate habitat affinities for some beetle groups.

A number of studies have shown that invertebrate species richness is correlated with plant species richness (Siemann et al., 1998; Beals, 2006; Schaffers et al., 2008). The high plant diversity of terra firme leads one to initially assume that terra firme habitat has more food resources than that of the neighboring bamboo forest, which may increase the number of herbivorous insects and other animals. The higher levels of plant-herbivore diversity could in turn increase animal diversity in higher trophic levels (Hunter & Price, 1993; Brose, 2003). Perhaps the fact that terra firme forest has more than double the number of tree species compared to adjacent bamboo-dominated forests (Griscom, Daly & Ashton, 2007) accounts at least in part, for why we found more beetle species and individuals in terra firme (especially for Carabidae and Curculionidae). However, our results show that overall beetle species richness from terra firme is only 17.5% greater than that of bamboo forest—a proportional difference that, according to the hypothesis above, is smaller than would be expected based on tree diversity patterns. Though we did not inventory tree species in our sample sites, our results suggest that factors other than plant diversity are contributing to the relatively high diversity of beetles in bamboo forests.

For beetles in the families Carabidae and Curculionidae, which are not often associated with dung and carrion, mechanistic hypotheses are more difficult. However, of our four target beetle families, these two families may be most strongly responding to the higher diversity of trees in the terra firme compared to bamboo forest. The majority of Neotropical carabid diversity occurs in the canopy of trees and the higher tree diversity of the terra firme may help explain the greater diversity of carabid beetles collected in this forest type. In addition, many curculionid beetles are plant, seed, or humus eaters, and thus may also be responding to the higher tree diversity of the terra firme. Although we collected a greater number of species of these two families in the terra firme, there was nonetheless high turnover in species composition between bamboo and terra firme.

### Seasonality and forest type

Seasonality alone is playing a role in structuring beetle communities, though the effects are mixed for different beetle families. Overall, we found more species in the wet season and more individuals in the dry season. Differences in beetle community structure between bamboo and terra firme were more pronounced in the wet season. Additionally, terra firme exhibited greater seasonal variation in the number of individuals captured and greater species turnover between seasons. Seasons in southeastern Peru are primarily defined by fluctuations in rainfall, and it is often assumed that increased rainfall will drive an increase in observed richness resulting from increased activity of most insect species (Wolda, 1978; Novotny & Basset, 1998).

We captured 35% more species in the wet season compared to the dry season, though many of the additional wet season species were singletons (i.e., the long tail in the species abundance distribution in Fig. 6B). Based on previous studies (Pearson & Derr, 1986; Devries, Walla & Greeney, 1999; Richards & Windsor, 2007), we also expected to observe higher abundances in the wet season versus the dry season. Interestingly, we captured 10% more individuals in the dry season compared to the wet season. However, it is important to acknowledge that the observed pattern of higher beetle abundance in the dry season is being driven primarily by scarab and histerid beetles—the most abundant families in our study. For the family Carabidae, 84% of all individuals were captured in the wet season. The abundance of curculionid beetles was approximately equal across dry and wet seasons.

While examining the effects of both seasonality and forest type on beetle community structure, our results, along with those from recent studies (Lucky, Erwin & Witman, 2002; Grimbacher & Stork, 2009), illustrate that temporal patterns of insects may be more complex than originally assumed. Overall, we found that season had a slightly stronger influence than forest type on beetle community structure when simultaneously analyzing the effects of habitat and season. However, when comparing beetle community structure in bamboo and terra firme separately for the dry and wet seasons, we observed that community structure was more highly defined in the wet season compared to the dry season.

In addition, there was more variation in the number of individuals captured, and greater species turnover, between the dry and wet seasons in terra firme compared to bamboo forest. Interestingly, while we found a larger difference in the number of individuals collected between dry and wet seasons in the terra firme, there was not a clear directional pattern. Thus, we collected a greater number of individuals at some sites in the wet season and a greater number of individuals at other sites in the dry season. In contrast, species richness was clearly higher in the wet versus the dry season for terra firme compared to bamboo forest (Figs. 9A and 9B). It is possible that bamboo forest is a more “predictable” habitat than terra firme in terms of food resources. Because terra firme has a much higher diversity of plants than bamboo forest, greater fluctuations in food resources from varying flowering and fruiting phenologies may occur throughout the year. Populations of beetle species that rely directly or indirectly on plant fruiting phenologies may be affected by this variation in terra firme. The potentially higher variation in beetle activity may explain why we observed more fluctuation in the number of individuals captured, and greater species turnover, between dry and wet seasons in terra firme. In contrast, bamboo plants from the genus *Guadua* do not experience the same annual phenological fluctuations compared to other tree species because they flower and set seed approximately every 30 years (Griscom, Daly & Ashton, 2007).

**Figure 11:**
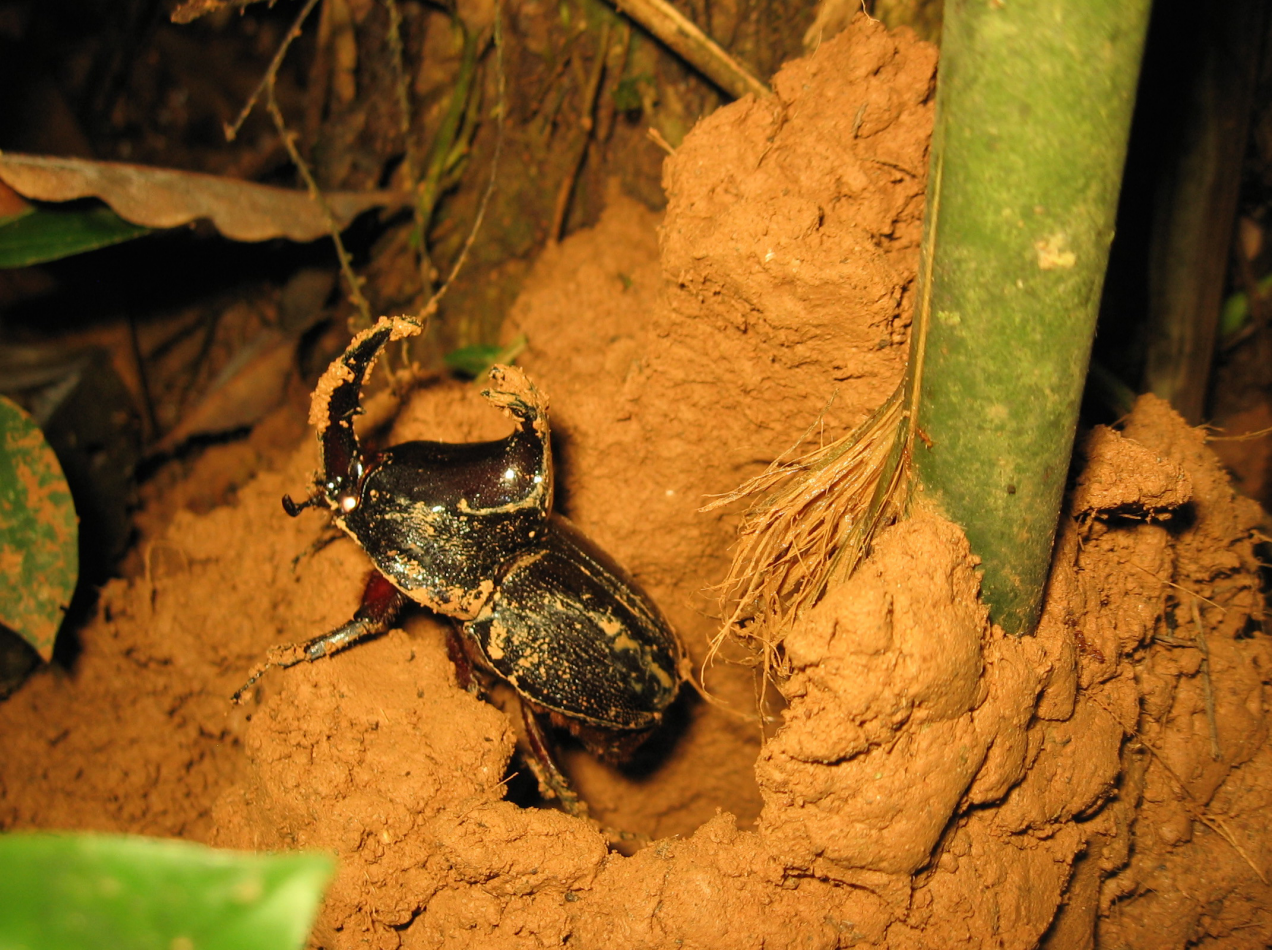
Individual of male *Enema pan* perching outside of his mound entrance at the base of a *Guadua weberbaueri* stem. *Enema pan* beetles construct these mounds connecting underground to approximately 1-meter long tunnels. When the mound is closed during the day, they stay hidden in their tunnels and feed on bamboo sap through the bark that they have shredded at the base of the bamboo stem. They open their mounds at night and perch at the entrance of their mound, presumably looking for mates or guarding the entrances to the mound. Photograph by Jennifer M. Jacobs.

Our results show that many beetle species are sensitive to seasonality and forest type. While a handful of beetle species were equally abundant in bamboo and terra firme, across wet and dry seasons, many were not. Other species, such as *Enema pan*, exhibited a strong preference for one forest type in only one season (Online Resource 1). After conducting additional investigations of *E. pan*, we discovered that this species lives in close association with the bamboo plants (Jacobs, von May & Ratcliffe, 2012). These results are important in that this species is possibly impacting bamboo plants through feeding and nesting damage, and because of their large size (∼100 mm), is potentially contributing considerably to the biomass of bamboo forest fauna (Fig. 11). In addition to *E. pan*, we found eight species of beetles that were captured in much greater numbers in the bamboo forest versus terra firme, suggesting that these species may also be associated with bamboo forest habitat (Online Resource 1). However, their biology is unknown. Bamboo-associated organisms are being discovered with increasing frequency (Jacobs & von May, 2012) and more in-depth field work, throughout the dry and wet seasons, is needed to determine if and why particular insect species are associated with specific forest types. Understanding insect diversity patterns in *Guadua* bamboo forests is particularly important because insects are the food source for so many other animals, and this is especially true for the 19 known species of insectivorous, *Guadua*-specialist birds (Kratter, 1997; Socolar, Robinson & Terborgh, 2013).

## Conclusions

*Guadua* bamboo forests are strikingly unique habitats because they occur as mono-dominant forest islands surrounded by hyper-diverse rainforest–providing an uncommon, spatially and biologically distinct habitat. Results from our study clearly show that bamboo forests in Peru maintain different communities of beetles compared to those of adjacent terra firme. Out of the total number of species that we collected in bamboo forest, 40% were collected only in bamboo forest. In addition, our results suggest that the species richness of beetles in terra firme is not considerably higher (17%) than that of bamboo forest–a surprising result given the substantially higher tree diversity in terra firme. Historically, bamboo forests in southwestern Amazonia have been regarded as species-poor, weedy habitats because of their relatively low plant diversity. It is encouraging to see that more research efforts are focusing on this important component of western Amazonian ecosystems. Bamboo-associated animal communities require more exploration and study, and must be included in regional conservation plans seeking to protect entire animal communities in southwestern Amazonia. Furthermore, with increased interest in harvesting natural bamboo or creating bamboo plantations for alternative timber sources, naturally-occurring bamboo forests may be at risk. In southwestern Amazonia, *Guadua* bamboo forests are also threatened by deforestation due to agriculture, illegal mining, and illegal logging. Thus, more studies focusing on the ecology of native bamboo forests and their associated fauna are of immediate and great importance.

##  Supplemental Information

10.7717/peerj.5153/supp-1 Data S1 Database (raw data) Database containing the following information: Family, family membership; SpN, name of species or morphospecies; Site, name of study site (each site contained one patch per habitat); Habitat, forest type (b, bamboo; tf, terra firme); Full_Site_Name, site name and habitat name; Trap, number of pitfall trap; Season, season (w, wet, d, dry); Abundance, number of individuals (each row represents one individual capture).

10.7717/peerj.5153/supp-2 Supplemental Information 1 Supplementary Material Table S1 and Table S2

## Additional Information and Declarations

### Field Study Permissions

The following information was supplied relating to field study approvals (i.e., approving body and any reference numbers): Permits were issued by the Instituto Nacional de Recursos Naturales (INRENA), Peru (Research authorizations 053-2005-INRENA-IFFS-DCB, 23-2006-INRENA-IFFS-DCB, 67- 2007-INRENA-IFFS-DCB, and 11-2008-INRENA-IFFS).

### Data Availability

The following information was supplied regarding data availability: The raw data are provided in the Supplemental Files.
