# Supplementary material for: Beetles in bamboo forests: community structure in a heterogeneous landscape of southwestern Amazonia
Source: PeerJ. 2018 Jul 3;6:e5153. doi: 10.7717/peerj.5153 (PMC6034595; doi:10.7717/peerj.5153)
Supplement: Supplemental Information 1 — Table S1 and Table S2 [file peerj-06-5153-s002.docx]

**Supplementary Material**

**Beetles in bamboo forests: community structure in a heterogeneous landscape of southwestern Amazonia**

Jennifer M. Jacobs^1,4^, Rudolf von May^2^, David H. Kavanaugh^3^, Edward F. Connor^1^

^1^ Department of Biology, San Francisco State University, 1600 Holloway Avenue, San Francisco, CA 94132

^2^ Museum of Zoology, Department of Ecology and Evolutionary Biology, University of Michigan, 2039 Ruthven Museums Building, Ann Arbor, MI 48109-1048

^3^ Department of Entomology, California Academy of Sciences, 55 Music Concourse Drive, San Francisco, CA 94118

^4^ Corresponding author; e-mail: jmjacobs.berkeley@gmail.com

**Table S1.**  Estimated patch area (ha), number of traps, and GPS coordinates (in decimal degrees) of the trap arrays used in this study.

| **Site** | **Habitat** | **Area (ha)** | **Nbr. of Traps** | **Longitude** | **Latitude** |
| --- | --- | --- | --- | --- | --- |
| Plataforma | Bamboo | 2 | 2 | -70.10987 | -12.55432 |
|  | Terra Firme |  | 2 | -70.11017 | -12.55440 |
| Cicra | Bamboo | 25 | 25 | -70.08888 | -12.55874 |
|  | Terra Firme |  | 25 | -70.09295 | -12.56241 |
| Daniela | Bamboo | 2 | 2 | -70.10714 | -12.55737 |
|  | Terra Firme |  | 2 | -70.10707 | -12.55759 |
| Huangana 2 | Bamboo | 2 | 2 | -70.14217 | -12.54284 |
|  | Terra Firme |  | 2 | -70.14176 | -12.54293 |
| Huangana | Bamboo | 9 | 9 | -70.14352 | -12.54207 |
|  | Terra Firme |  | 9 | -70.14448 | -12.54158 |
| Huanganita | Bamboo | 2 | 2 | -70.12718 | -12.54884 |
|  | Terra Firme |  | 2 | -70.12694 | -12.54893 |
| Jean | Bamboo | 6 | 6 | -70.09909 | -12.56540 |
|  | Terra Firme |  | 6 | -70.09883 | -12.56571 |
| Luisa 2 | Bamboo | 1 | 1 | -70.11514 | -12.54080 |
|  | Terra Firme |  | 1 | -70.11495 | -12.54060 |
| Luisa | Bamboo | 7 | 7 | -70.11702 | -12.54224 |
|  | Terra Firme |  | 7 | -70.11739 | -12.54257 |
| Maderero | Bamboo | 5 | 5 | -70.11495 | -12.54581 |
|  | Terra Firme |  | 5 | -70.11549 | -12.54582 |
| Pacal | Bamboo | 7 | 7 | -70.13425 | -12.54241 |
|  | Terra Firme |  | 7 | -70.13502 | -12.54289 |
| Palmeras | Bamboo | 8 | 8 | -70.10424 | -12.54373 |
|  | Terra Firme |  | 8 | -70.10484 | -12.54407 |
| Sobrevuelo | Bamboo | 3 | 3 | -70.10767 | -12.56000 |
|  | Terra Firme |  | 3 | -70.10751 | -12.55969 |

**Table S2.**  A list of all species captured indicating in which forest type they were found and in which season. “B” represents bamboo forest, “TF” represents terra firme forest, “Dry” represents the dry season, and “Wet” represents the wet season. Those species marked with an * may be strongly associated with bamboo forest based on their observed patterns of relative abundance in the two forest types. The species list for Curculionidae is numbered for morpho-species because, while we had help from a taxonomist with identifications, all of the species we collected were undescribed or unknown to the specialist.

| **Taxa** | **Taxonomic authority** | **B** | **TF** | **Dry** | **Wet** |
| --- | --- | --- | --- | --- | --- |
|  |  |  |  |  |  |
| **Carabidae** | Latreille, 1802 |  |  |  |  |
| *Abaris erwini* | Will, 2002 | 0 | 1 | 1 | 0 |
| *Apenes A* | LeConte, 1851 | 1 | 0 | 1 | 0 |
| *Apenes B* | LeConte, 1851 | 1 | 0 | 1 | 0 |
| *Apenes C* | LeConte, 1851 | 0 | 1 | 1 | 0 |
| *Coptodera A* | Dejean, 1825 | 0 | 1 | 1 | 0 |
| *Dercylus A* | Castelnau de Laporte, 1832 | 2 | 0 | 0 | 2 |
| *Dercylus B* | Castelnau de Laporte, 1832 | 1 | 0 | 0 | 1 |
| *Galerita jelskii* | Fabricius, 1801 | 17 | 7 | 2 | 22 |
| *Glyptogrus A* | Bates, 1881 | 5 | 9 | 2 | 12 |
| *Helluobrochus A* | Reichardt, 1974 | 0 | 1 | 1 | 0 |
| *Loxandrus A* | LeConte, 1852 | 1 | 1 | 0 | 2 |
| *Loxandrus B* | LeConte, 1852 | 0 | 1 | 1 | 0 |
| *Notiobia A* | Perty, 1830 | 1 | 0 | 0 | 1 |
| *nr. Loxandrus* | Latreille, 1802 | 0 | 2 | 0 | 2 |
| *Nyctostyles A* | Putzeys, 1866 | 5 | 1 | 0 | 6 |
| *Odontocheila annulicornis* | Brulle, 1837 | 0 | 1 | 0 | 1 |
| *Odontocheila cayennensis* | Fabricius, 1787 | 14 | 17 | 3 | 28 |
| *Odontocheila rufiscapis* | Bates, 1874 | 0 | 2 | 0 | 2 |
| *Pachyteles nr. striola* | Perty, 1830 | 1 | 0 | 0 | 1 |
| *Pseudabarys A* | Chaudoir, 1873 | 0 | 1 | 0 | 1 |
| *Rhysodina A* | Castlenau, 1840 | 0 | 1 | 0 | 1 |
| *Scarites A* | Fabricius, 1775 | 1 | 0 | 0 | 1 |
| *Selenophorus A* | Dejean, 1831 | 1 | 2 | 2 | 1 |
| *Stratiotes A* | Putzeys, 1846 | 0 | 1 | 0 | 1 |
| *Tichonilla A* | Strand, 1942 | 0 | 2 | 0 | 2 |
|  |  |  |  |  |  |
| **Histeridae** | Gyllenhal, 1808 |  |  |  |  |
| *Conchita propygidiale* | Mazur, 1994 | 1 | 0 | 1 | 0 |
| *Euspilotus A* | Lewis, 1907 | 0 | 1 | 0 | 1 |
| *Euspilotus B* | Lewis, 1907 | 1 | 0 | 1 | 0 |
| *Exosternini A* | Bickhardt, 1914 | 0 | 1 | 0 | 1 |
| *Exosternini B* | Bickhardt, 1914 | 1 | 2 | 3 | 0 |
| *Exosternini C* | Bickhardt, 1914 | 0 | 1 | 1 | 0 |
| *Exosternini D* | Bickhardt, 1914 | 4 | 7 | 10 | 1 |
| *Omalodes A* | Dejean, 1833 | 61 | 93 | 54 | 100 |
| *Omalodes B* | Dejean, 1833 | 2 | 0 | 2 | 0 |
| *Operclipygus kerga* | Caterino & Tishechkin, 2013 | 1 | 0 | 0 | 1 |
| *Operclipygus inflatus* | Caterino & Tishechkin, 2013 | 0 | 2 | 0 | 2 |
| *Operclipygus foveipygus** | Bickhardt, 1918 | 43 | 11 | 47 | 7 |
| *Operclipygus fossipygus* | Wenzel, 1944 | 4 | 3 | 6 | 1 |
| *Operclipygus hospes* | Lewis, 1902 | 7 | 2 | 4 | 5 |
| *Operclipygus impuncticollis* | Hinton, 1935 | 3 | 2 | 2 | 3 |
| *Operclipygus lama* | Mazur, 1988 | 1 | 2 | 2 | 1 |
| *Operclipygus juninensis* | Caterino & Tishechkin, 2013 | 3 | 2 | 3 | 2 |
| *Paratropus* | Gerstaecker, 1867 | 0 | 5 | 0 | 5 |
| *Phelister A* | Marseul, 1853 | 2 | 0 | 2 | 0 |
| *Phelister B* | Marseul, 1853 | 18 | 92 | 64 | 46 |
| *Phelister blairi* | Hinton, 1935 | 1 | 1 | 1 | 1 |
| *Scapomegas auritus* | Marseul, 1855 | 0 | 1 | 1 | 0 |
|  |  |  |  |  |  |
| **Scarabaeidae** |  |  |  |  |  |
| *Anaides onofrii* | Ocampo, 2006 | 4 | 0 | 4 | 0 |
| *Anomiopus D* | Westwood, 1843 | 4 | 1 | 0 | 5 |
| *Anomiopus F* | Westwood, 1843 | 2 | 1 | 1 | 2 |
| *Aphodiinae A* | Leach, 1815 | 0 | 4 | 0 | 4 |
| *Aphodiinae B* | Leach, 1815 | 1 | 0 | 0 | 1 |
| *Ateuchus A* | Weber, 1801 | 0 | 1 | 0 | 1 |
| *Ateuchus C* | Weber, 1801 | 0 | 102 | 14 | 88 |
| *Ateuchus D* | Weber, 1801 | 18 | 50 | 53 | 15 |
| *Ateuchus F* | Weber, 1801 | 0 | 15 | 5 | 10 |
| *Ateuchus H* | Weber, 1801 | 3 | 0 | 2 | 1 |
| *Ateuchus P* | Weber, 1801 | 0 | 1 | 1 | 0 |
| *Athyreus A* | MacLeay, 1819 | 0 | 1 | 0 | 1 |
| *Bdelyrus parvus* | Cook, 1998 | 1 | 0 | 0 | 1 |
| *Canthidium A* | Erichson, 1847 | 64 | 104 | 115 | 53 |
| *Canthidium batesi* | Harold, 1867 | 11 | 72 | 0 | 83 |
| *Canthidium bicolor* | Boucomont, 1928 | 1 | 2 | 2 | 1 |
| *Canthidium cf. AA* | Erichson, 1847 | 0 | 4 | 0 | 4 |
| *Canthidium cupreum* | Blanchard, 1846 | 1 | 2 | 3 | 0 |
| *Canthidium F* | Erichson, 1847 | 99 | 65 | 84 | 80 |
| *Canthidium G* | Erichson, 1847 | 0 | 1 | 1 | 0 |
| *Canthidium gerstaeckeri* | Harold, 1867 | 75 | 115 | 57 | 133 |
| *Canthidium histrio* | Balthasar, 1939 | 0 | 1 | 0 | 1 |
| *Canthidium nr. deyrollei* | Erichson, 1847 | 32 | 123 | 32 | 123 |
| *Canthidium nr. dohrni* | Erichson, 1847 | 0 | 7 | 0 | 7 |
| *Canthidium nr. funebre* | Erichson, 1847 | 3 | 0 | 0 | 3 |
| *Canthidium nr. histrio* | Erichson, 1847 | 7 | 6 | 7 | 6 |
| *Canthidium nr. kiesenwetteri* * | Erichson, 1847 | 48 | 3 | 23 | 28 |
| *Canthidium nr. quadridens* | Erichson, 1847 | 74 | 74 | 94 | 54 |
| *Canthidium nr. smaragdinum* | Erichson, 1847 | 2 | 0 | 0 | 2 |
| *Canthidium P* | Erichson, 1847 | 1 | 0 | 0 | 1 |
| *Canthidium T* | Erichson, 1847 | 2 | 7 | 2 | 7 |
| *Canthon A* | Hoffmannsegg, 1817 | 5 | 139 | 126 | 18 |
| *Canthon aequinoctialis* | Harold, 1868 | 1 | 2 | 3 | 0 |
| *Canthon femoralis bimaculatus* | Schmidt, 1922 | 0 | 6 | 6 | 0 |
| *Canthon luteicollis* | Erichson, 1847 | 16 | 4 | 18 | 2 |
| *Canthon nr. angustatus* | Erichson, 1847 | 0 | 19 | 16 | 3 |
| *Canthon semiopacus* | Harold, 1868 | 3 | 4 | 7 | 0 |
| *Canthon sericatus* | Schmidt, 1922 | 2 | 3 | 3 | 2 |
| *Canthonella B* | Chapin, 1930 | 6 | 0 | 2 | 4 |
| *Canthonella D* * | Chapin, 1930 | 55 | 10 | 28 | 37 |
| *Ceratocanthinae A* | Martínez, 1968 | 9 | 14 | 0 | 23 |
| *Ceratocanthinae B* | Martínez, 1968 | 8 | 1 | 3 | 6 |
| *Chaetodus A* * | Westwood, 1845 | 59 | 3 | 27 | 35 |
| *Chaetodus B* | Westwood, 1845 | 2 | 0 | 1 | 1 |
| *Coprophanaeus cf. parvulus* | Olsoufieff, 1924 | 0 | 1 | 0 | 1 |
| *Coprophanaeus lancifer* | Linnaeus, 1767 | 0 | 4 | 0 | 4 |
| *Coprophanaeus telamon* | Erichson, 1847 | 0 | 1 | 0 | 1 |
| *Cryptocanthon campbellorum* | Howden, 1973 | 4 | 6 | 8 | 2 |
| *Deltochilum amazonicum* | Bates, 1887 | 1 | 0 | 1 | 0 |
| *Deltochilum barbipes grp. B* | Bates, 1870 | 0 | 9 | 8 | 1 |
| *Deltochilum carinatum* | Westwood, 1837 | 0 | 1 | 1 | 0 |
| *Deltochilum granulatum* * | Bates, 1870 | 41 | 7 | 42 | 6 |
| *Deltochilum howdeni* | Martinez, 1955 | 1 | 8 | 3 | 6 |
| *Deltochilum laevigatum* | Paulian, 1938 | 5 | 7 | 6 | 6 |
| *Deltochilum nr. komerecki* | Eschsholtz, 1822 | 3 | 33 | 17 | 19 |
| *Deltochilum valgum* | Burmeister, 1873 | 0 | 3 | 1 | 2 |
| *Dendropaemon nr. faces* | Perty, 1830 | 0 | 1 | 0 | 1 |
| *Dichotomius batesi* * | Harold, 1869 | 79 | 1 | 37 | 43 |
| *Dichotomius lucasi* | Harold, 1869 | 30 | 98 | 98 | 30 |
| *Dichotomius nr. fonsecae* | Hope, 1838 | 0 | 8 | 0 | 8 |
| *Dichotomius nr. lucasi* | Hope, 1838 | 34 | 38 | 51 | 21 |
| *Dichotomius ohausi* | Luederwaldt, 1923 | 59 | 100 | 33 | 126 |
| *Dichotomius sp. A* | Hope, 1838 | 0 | 2 | 0 | 2 |
| *Dynastinae B* | MacLeay, 1819 | 1 | 0 | 0 | 1 |
| *Enema pan* * | Fabricius, 1775 | 63 | 5 | 0 | 68 |
| *Eurysternus caribaeus* | (Herbst, 1879) | 3 | 14 | 13 | 4 |
| *Eurysternus hypocrita* | Balthasar, 1939 | 6 | 12 | 17 | 1 |
| *Eurysternus nov. strigilatus* | Génier, 2009 | 6 | 32 | 34 | 4 |
| *Eurysternus plebejus* | Harold, 1880 | 5 | 1 | 6 | 0 |
| *Hybosoridae C* | Erichson, 1947 | 1 | 0 | 0 | 1 |
| *Hybosoridae D* | Erichson, 1947 | 1 | 0 | 0 | 1 |
| *Melolonthinae A* | Samouelle, 1819 | 1 | 0 | 1 | 0 |
| *Melolonthinae B* | Samouelle, 1819 | 0 | 1 | 0 | 1 |
| *Melolonthinae C* | Samouelle, 1819 | 1 | 0 | 0 | 1 |
| *Ontherus raptor* | Génier, 1996 | 0 | 2 | 1 | 1 |
| *Onthophagus A* | Latreille, 1802 | 0 | 15 | 15 | 0 |
| *Onthophagus haematopus* | Harold, 1875 | 4 | 0 | 2 | 2 |
| *Onthophagus rhinophyllus* | Harold, 1868 | 3 | 1 | 4 | 0 |
| *Onthophagus xanthomerus* | Bates, 1887 | 69 | 154 | 207 | 16 |
| *Oxysternon spiniferum* | (Laporte, 1840) | 0 | 1 | 0 | 1 |
| *Phaneus cambeforti* | Arnaud, 1982 | 5 | 79 | 40 | 44 |
| *Phileurus didymus* | (Linnaeus, 1758) | 2 | 1 | 1 | 2 |
| *Scybalocanthon C* | Martínez, 1948 | 112 | 146 | 161 | 97 |
| *Scybalocanthon D* | Martínez, 1948 | 49 | 69 | 85 | 33 |
| *Scybalocanthon F* | Martínez, 1948 | 7 | 0 | 3 | 4 |
| *Scybalocanthon K* * | Martínez, 1948 | 36 | 1 | 14 | 23 |
| *Scybalocanthon zischkai* | Martínez, 1949 | 3 | 0 | 0 | 3 |
| *Sylvicanthon bridarollii* * | (Martínez, 1949) | 18 | 2 | 20 | 0 |
| *Uroxys D* | Westwood, 1843 | 0 | 9 | 7 | 2 |
| *Uroxys K* | Westwood, 1843 | 1 | 0 | 0 | 1 |
|  |  |  |  |  |  |
| **Curculionidae** | Latreille, 1802 |  |  |  |  |
| Curculionidae *1* |  | 0 | 7 | 0 | 7 |
| Curculionidae *2* |  | 1 | 7 | 0 | 8 |
| Curculionidae *3* |  | 1 | 2 | 0 | 3 |
| Curculionidae *4* |  | 2 | 3 | 0 | 5 |
| Curculionidae *5* |  | 1 | 1 | 0 | 2 |
| Curculionidae *6* |  | 0 | 1 | 0 | 1 |
| Curculionidae *7* |  | 0 | 1 | 1 | 0 |
| Curculionidae *8* |  | 6 | 0 | 3 | 3 |
| Curculionidae *10* |  | 2 | 1 | 1 | 2 |
| Curculionidae *11* |  | 0 | 1 | 0 | 1 |
| Curculionidae *12* |  | 0 | 1 | 0 | 1 |
| Curculionidae *13* |  | 6 | 8 | 10 | 4 |
| Curculionidae *14* |  | 18 | 19 | 37 | 0 |
| Curculionidae *15* |  | 0 | 1 | 0 | 1 |
| Curculionidae *16* |  | 0 | 2 | 0 | 2 |
| Curculionidae *17* |  | 0 | 3 | 3 | 0 |
| Curculionidae *18* |  | 1 | 0 | 0 | 1 |
| Curculionidae *19* |  | 0 | 1 | 0 | 1 |
| Curculionidae *20* |  | 1 | 1 | 1 | 1 |
| Curculionidae *21* |  | 4 | 0 | 2 | 2 |
| Curculionidae *22* |  | 0 | 2 | 1 | 1 |
| Curculionidae *23* |  | 2 | 2 | 0 | 4 |
| Curculionidae *24* |  | 2 | 0 | 0 | 2 |
| Curculionidae *25* |  | 0 | 2 | 0 | 2 |
| Curculionidae *26* |  | 1 | 0 | 0 | 1 |
| Curculionidae *27* |  | 0 | 1 | 0 | 1 |
| Curculionidae *28* |  | 0 | 1 | 1 | 0 |
| Curculionidae *29* |  | 0 | 2 | 1 | 1 |
| Curculionidae *30* |  | 0 | 2 | 0 | 2 |
| Curculionidae *31* |  | 0 | 1 | 0 | 1 |
| Curculionidae *32* |  | 0 | 5 | 0 | 5 |
| Curculionidae *33* |  | 0 | 2 | 0 | 2 |
| Curculionidae *34* |  | 1 | 0 | 0 | 1 |
| Curculionidae *35* |  | 2 | 3 | 0 | 5 |
| Curculionidae *36* |  | 1 | 2 | 1 | 2 |
| Curculionidae *37* |  | 0 | 1 | 0 | 1 |
| Curculionidae *38* |  | 1 | 0 | 0 | 1 |
| Curculionidae *39* |  | 1 | 0 | 1 | 0 |
| Curculionidae *40* |  | 0 | 1 | 1 | 0 |
| Curculionidae *41* |  | 0 | 2 | 0 | 2 |
| Curculionidae *42* |  | 1 | 0 | 0 | 1 |
| Curculionidae *43* |  | 1 | 0 | 1 | 0 |
| Curculionidae *44* |  | 0 | 1 | 0 | 1 |
| Curculionidae *45* |  | 0 | 1 | 0 | 1 |
| Curculionidae *46* |  | 1 | 0 | 1 | 0 |
| Curculionidae *47* |  | 0 | 1 | 0 | 1 |
| Curculionidae *48* |  | 1 | 0 | 1 | 0 |
| Curculionidae *49* |  | 1 | 0 | 0 | 1 |
| Curculionidae *50* |  | 1 | 0 | 1 | 0 |
| Curculionidae *51* |  | 1 | 0 | 1 | 0 |
| Curculionidae *52* |  | 1 | 0 | 1 | 0 |
| Curculionidae *53* |  | 0 | 1 | 1 | 0 |
